# An American Object Lesson

**Published:** 1889-06-01

**Authors:** 


### The Hospital

A Weekly Institutional Journal of

**Science, Medicine, Nursing, and Philanthropy.**

*For Hospitals, Asylums, Medical Practitioners, Students, Nurses, Families, Parishes, Congregations, and the Charitable Public.*

Vol. VI. No. 140.] [June 1, 1889.

### An American Object Lesson.

America herself has long been a huge object lesson. She has shown to kings and peoples for nearly two centuries what plain men and women can do in the subjugation of natural difficulties, the solution of state problems, the creation of a various and worthy literature, and the foundation and maintenance of a high type of national character. All this she has done and more. It is not, however, our present object to hold up America and Americans for general admiration, but rather to bring into clearer distinctness the special features of a great American institution, which will be described in fuller detail in another issue.

"We are on the verge of great advances in our knowledge of the causes and methods of disease, and I feel sure that these will be only preliminary steps to far greater and better knowledge of how to prevent or to treat them than we now have. The probable length of life of the new-born infant to-day is not much more than half what it ought to be: the practical productive period of the life of our men and women is shortened and interrupted by unnecessary disease and suffering. But, remember, if these things are to be amended it is not merely by teaching old doctrines; we must open fresh windows and let in more light, so that we can see what these obstacles really are. It is in this work of discovery that it is hoped that this hospital will join hands with the university, and it is in this hope that some of the structures around you have been planned and provided." Thus Dr. J. S. Billings, of the United States Army, a man of note in the medical world in his own and other countries, expressed himself on the occasion of the opening ceremony of the Johns Hopkins Hospital in Baltimore a few weeks ago.

To know the causes and methods of disease in order that we may have more and better knowledge of prevention and treatment: that is the note of the best physicians and surgeons of the present day. To prevent first of all if that be possible; and where prevention is not possible to treat rationally, and, as far as may be, successfully. The Johns Hopkins Hospital has been founded by the wisely-directed benevolence of one man, and it bears his honoured name. But it bears more than his name; it has also received the distinct impress of his reasonable and right-minded personality. Mr. Johns Hopkins was a Quaker. The members of the Society of Friends have great respect for duties clearly understood and well performed. The typical Quaker is, above all things, a clear-minded, duty-doing man or woman. But these are the very principles which lie at the root of the Baconian and every other rational method of science. To put prejudice away; to know a thing as well as it can be known, and to do it as well as it can be done—what is that but to move forward on the only lines on which any kind of progress is possible? That was Bacon's way, in theory; it was George Fox's way, in theory and practice, too; it is, and always has been, every man's way who has been endowed with strong intelligence and an authoritative conscience. It was Johns Hopkins's way, at least when he founded the hospital which bears his name.

The hospital is to be a place of treatment for the sick, both for those who can pay, and those who cannot. But it is to be also, and perhaps still more, a "school of medicine." Now, a "school of medicine" is not a mere workshop for the repairing of broken-down bodies. It is a place where the nature and constitution of man are to be studied; where the influences of all kinds of life conditions and environments are to be recorded and their value estimated; a place where knowledge is to be accumulated from which sound and large principles may be drawn—principles which shall not be hidden in case-books nor in the obscurities of the medical mind, but which shall direct individuals and states how to traverse the highways and byeways of life with victorious safety and prosperity. These and their like are the natural and proper functions of a great hospital.

"No attempts were made," says Dr. Billings, "to select and engage individual members of the hospital staff until quite recently; but there was, nevertheless, a tolerably definite conception as to the ideas, mode of work, character, and wants of those who are to constitute this staff, and when the time came for selecting it was made by this standard." It would be highly advantageous to know whether the greater proportion of the men selected possess the practical skill, and, above all, the philosophic breadth of mind and culture which will enable them worthily to carry out the large and liberal ideas of the founder of the hospital, and the equally large and liberal ideas which seem to possess the mind of Dr. Billings on this subject. If such men have been found and appointed, how have they been found and appointed? Those are the kind of men wanted for the great hospitals of this country—wanted, but how often vainly wanted! Skilled specialists we have by the score, even in hospitals the most obscure. But where are the hospital physicians, even in this metropolis of London, who, in addition to their technical skill, possess the statesman's large and liberal humanity, or the philosopher's cultured breadth? "Knowledge comes, but wisdom lingers,"

wrote Tennyson, and it is grievously true of the present age that specialism grows more and more, whilst broad humanity grows less and less." The doctor is a doctor, and must be a doctor and nothing else," is the dictum even of many of the Conscript Fathers of medicine in this country. So saying, and so acting, they degrade medicine from the lofty eminence of a science of man, and make her nothing else but a mere mechanical repairer of mechanical injuries. To know how to prevent all manner of diseases and ills, and to spread your knowledge of prevention into every nook and corner of the world, so that all human life may be stronger, longer, healthier, and happier; that is the highest ideal which modern medicine can set before itself, and any lower ideal ought not even to be named by the members of a humane profession.

Rational and skilled treatment is the second note of modern medicine, as prevention is the first. Treatment must be skilled in detail, and, where necessary, continuous. In many cases the doctor's visit and instructions would be the most miserable futilities if not followed up by the constant application of his principles in hourly practice. The trained nurse is thus a necessity. She is the resident physician, so to speak, acting under the authority of the visiting physician. "I can only say," says Dr. Billings, "that in many cases a competent trained nurse is as important to the success of the treatment as a competent doctor, and that one of the greatest difficulties in treating well-to-do patients in their own homes in this city is the want of proper nurses." For the purpose of training such nurses the trustees of the Johns Hopkins Hospital "have provided a large and handsome building, separated from the others, and exclusively appropriated to the female nurses, where each can have her own comfortable room, and where a common parlour, library, dining-room, bathrooms, and, in short, the arrangements of a first-class hotel, are provided for their use. . . . The intention is that when the nurse has finished her six or eight hours' tour of duty with the sick she shall come quite away from the ward and all that pertains to it, and take her rest and recreation in a totally different atmosphere, and special efforts have been made to have this home attractive and pleasant." This is in accordance with the dictates both of science and sense, and is of a piece with the whole of the highly-intelligent policy which has guided the founder and his lieutenants throughout.

So far as appears at present, the Johns Hopkins Hospital promises to be a model of what such institutions ought to be. The story of its origin in the founder's mind, and of its birth struggles in the architect stage, as well as of its after growth and its final completion, is told by Dr. Billing with admirable lucidity and enthusiasm. A prose epic was wanted for the occasion, and, perhaps, no man in the United States, not excepting Holmes and Lowell themselves, could have so well and suitably matched large and generous ideas with liberal and glowing words. It is fervently to be hoped that the great and admirable objects contemplated by the founder may be the constant inspiration of those who administer the hospital in all its parts; and that a noble humanity allied with a progressive science may animate its work from the first day of its existence to the last.

